# Comparison of the Novel Thin Film-Solid Phase Microextraction and Sorptive Extraction Methods for *Picual* and *Hojiblanca* Olive Oil Volatile Fraction Analysis in Headspace

**DOI:** 10.3390/foods9060748

**Published:** 2020-06-05

**Authors:** M. Pilar Segura-Borrego, Rocío Ríos-Reina, Cristina Ubeda, Raquel M. Callejón, M. Lourdes Morales

**Affiliations:** Área de Nutrición y Bromatología, Dpto. de Nutrición y Bromatología, Toxicología y Medicina Legal, Facultad de Farmacia, Universidad de Sevilla, C/P, García González n°2, E-41012 Sevilla, Spain; msegura2@us.es (M.P.S.-B.); rrios5@us.es (R.R.-R.); c_ubeda@us.es (C.U.); mlmorales@us.es (M.L.M.)

**Keywords:** extra virgin olive oil, thin film, twister-polydimethylsiloxane, volatile compound, PARADISe software

## Abstract

For first time, the new device named thin film solid phase microextraction (TF-SPME) has been used to determine the volatile profile of the *Picual* and *Hojiblanca* varieties of extra virgin olive oils. To this end, different traditional sampling methods such as headspace sorptive extraction (HSSE) with polydimethylsiloxane (PDMS) and polyethyleneglycol-modified silicone (EG/Silicone) Twisters^®^ have been compared with the TF-SPME devices coated with different extraction polymeric phases. PARADISe software was used as a non-targeting method to process all data. The best results were obtained by HSSE-PDMS and 2TF-SPME. Moreover, the 2TF-SPME extraction method achieved the most adequate results of linearity for most compounds, according to F-values, while the intermediate precision results were similar for both 2TF-SPME and HSSE-PDMS sampling methods. Different sensitivity was observed between both sampling methods depending on the volatile compound, without being clearly influenced by the polarity of them. Although both sampling methods enabled the main active aroma of olive oil to be determined and for them to be differentiated according to olive variety, the 2TF-SPME method appears to be the most suitable for this goal.

## 1. Introduction

Extra virgin olive oil (EVOO) is an olive oil with an absence of sensory defects obtained from the fresh and healthy fruit of the olive tree, *Olea Europe L.* [1,2,3]. European legislation establishes that EVOO is characterised by a positive evaluation given from a sensory panel as well as by physicochemical parameters, such as acidity, that can determine its quality [4].

EVOO is obtained from olives by using mechanical and other physical methods when the fruit is in its optimum phase of ripeness [1,5] and can be consumed without any further refining [2,3].

The European Union (EU) is the world’s main olive oil producer, accounting for 80% of the total world production. In terms of crop surface and volume of olive oil production, Spain is world leader, accounting for approximately 60% of EU production and 45% of world production. Spain is also the leading global exporter of olive oil [6].

Nowadays, EVOO is a product that is highly appreciated by consumers. It is well-known that regular dietary consumption of EVOO has health benefits. However, it is not only appreciated for this reason; EVOO is also a vegetable oil with excellent sensory aroma, thanks to its volatile compounds. Indeed, aroma is one of the main quality indicators of any food. The characteristic volatile compounds of EVOO are determined by different factors: the effects of climate, soil, geographic origin, cultivar of the olives, and degree of ripeness of the fruit or its storage conditions [1,7]. According to the literature, EVOO aroma is constituted by a large number of volatile compounds that mainly belong to chemical groups: alcohols, esters, aldehydes, ketones, furans, hydrocarbons and other as yet unidentified groups [8]. The main precursors of volatile compounds are fatty acids [1,2,7,8,9]. During the oil extraction process, the action of endogenous enzymes forms these volatile compounds by degrading polyunsatured fatty acids. This process, therefore, is one of the factors responsible for the positive aroma perceptions of EVOO [1,7].

Many reasons, such as growing consumer demand, the increasing diversity of EVOO, the high quality of such oils and the high price in the market, have led to the need to know their composition and characterise their aroma, enabling them to be differentiated, thus providing a suitable means of quality control in order to protect them against fraud. Although sensory analysis is widely-used for these purposes, as a tool that enables a quick evaluation of organoleptic quality of olive oil, the results of a recent European project have pointed out that there are disagreements between different taster panels. Therefore, the sensory test of olive oil requires a globally standardized procedure supported by suitable methods to determine volatile profile, especially in cases of confirmatory analyses or of disagreement between panels [10].

Gas chromatography-mass spectrometry (GC/MS) has been the most widely-used technique for analysing the volatile compounds of olive oil. Furthermore, a prior extraction is needed in order to perform these analyses. As a result, different extraction techniques have been applied for the analysis of the volatile composition in EVOO, such as: dynamic headspace (DHS) [11], direct thermal desorption (DTD) [12], simultaneous distillation/extraction (SDE) [13], closed-loop stripping analysis (CLSA) [13], static headspace (SHS) [12], solid phase microextraction (SPME) [11,12,13,14] and stir bar into headspace (HSSE) [12,13]. According to previous comparative works, the most complete knowledge of the volatile profile has been achieved by SPME and HSSE. Both are solvent-free sampling techniques and therefore, according to the new trend, are termed Green Chemistry.

On the one hand, SPME has been employed for a rapid and accurate olive oil aroma characterisation [10,12,14]. Among the various fibres that exist for SPME, divinylbenzene/carboxen/polydimethylsiloxane (DVB/CAR/PDMS) has been the most widely-used stationary phase for extracting the volatile compounds in olive oil [15]. This triple-phase fibre enables compounds with different polarities to be extracted. However, the main drawback of SPME is the low amount of polymer covering the fibre, thus affecting sensitivity [16]. For this reason, new SPME devices have been developed, such as SPME Arrows or the new Thin Film (TF-SPME) that have a large surface of extraction phase, which increases the technique’s sensitivity. In fact, the first one has been demonstrated to be ten times more sensitive than traditional SPME fibres [17], favouring the enrichment of polar compounds in the extraction. Moreover, it is reusable and therefore the per-analysis cost [18,19] is lowered. Both devices have been used to determine volatile compounds in different food matrices, such as SPME arrows in sauce [20] and TF-SPME in beer [19] and other beverages [21], but they have never been used previously in an olive oil matrix.

HSSE is a technique based on the sorption of analytes on a device named Twister^®^. Twister^®^ is a stir bar commonly coated with polydimethylsiloxane (PDMS), a non-polar phase. Several advantages in the use of Twister^®^ have been described, including rapid thermal desorption at mild temperatures, the absence of displacement effects, inertness, predictable enrichment and a sensitivity a thousand times greater than SPME, due to its greater volume of sorbent [22,23]. In addition, Twister^®^ can perform the extraction in the headspace (HSSE), reducing the risk of contamination and increasing the stir bar’s lifetime. However, one of the disadvantages is the low extraction rate of polar volatile compounds due to the coating phase (PDMS) of Twister^®^. For this reason, in recent years, new Twister^®^ stir bars coated with a higher polarity phase, such as polyethyleneglycol-modified silicone (EG/S), have been marketed in order to improve the extraction of polar volatile compounds [24,25,26].

The EVOO volatile profile obtained by HSSE-GC/MS and SPME-GC/MS analysis is a complex dataset, and its treatment is lengthy. Recent research works have used different kinds of software packages to process GC/MS datasets by a non-targeted approach such as Deconvolution and Identification System (PARADISe), because it helps to solve some problems, such as the co-elution of the peaks. PARADISe enables data with a good resolution to be obtained, and it enables chemical information to be extracted directly from the raw data [27]. PARADISe is a powerful methodology for analysing complex chromatographic data created and described by Johnsen et al. [27]. PARADISe software, based on Parallel Factor Analysis 2 (PARAFAC2), is an independent, freely available computer platform software that incorporates a number of newly-developed algorithms in a coherent framework. The theory of PARADISe allows the simultaneous deconvolution of the pure mass spectra of peaks, and the integration of areas of deconvoluted peaks for all samples; resolved peaks are identified using their deconvoluted pure mass spectra [27].

In this context, the aim of the present work was to apply the new TF-SPME devices to determine the volatiles composition of *Picual* and *Hojiblanca* variety EVOOs for the first time, and to assess the suitability of this extraction technique compared with HSSE techniques, as an extraction method with a proven efficiency.

## 2. Materials and Methods

### 2.1. Samples

Two extra virgin olive oils from *Hojiblanca and Picual*, two olive varieties native to Spain, were studied. These samples were provided by Aceite Supermo S.L. (Jaén, Spain). These olive oils were produced in Jaen using olives from this production area.

In addition to the aforementioned samples, a refined olive oil provided by ACESUR, ‘Aceites del Sur S.L.’ was used to perform the linearity, sensitivity and intermediate precision assays.

### 2.2. Chemicals and Materials

Alkane standard mixture C_10_–C_40_, used for calculating the Linear Retention Index (LRI), was purchased from Fluka (Madrid, Spain). Milli-Q water was obtained from a Milli-Q purification system (Millipore, Burlington, MA, USA).

The microextraction devices used to extract the volatile fraction were: polydimethylsiloxane (PDMS) Twister^®^; polyethyleneglycol-modified silicone (EG/S) Twister^®^; and the recently-developed thin film solid phase microextraction (TF-SPME), with two different kinds of coating, divinylbenzene/polydimethylsiloxane coating (DVR/PDMS) or carboxen/polydimethylsiloxane (CAR/PDMS) as the extraction phase. All microextraction devices were purchased from Gerstel (Müllheim an der Ruhr, Germany). The PDMS Twister^®^ length was 10 mm, with a 24 µL coating; the length of EG/S Twister^®^ was 10 mm, with a 32 µL coating; the TF-SPME device was a 20 × 4.8 mm carbon mesh sheet impregnated with a coating phase. All devices were previously conditioned following the supplier’s instructions.

### 2.3. Volatile Fraction Extraction Methods

All extraction procedures were performed in sample headspace in duplicate. The sampling devices tested were: Twister^®^, with two different kind of coating phases (PDMS and EG/S) and two kinds of TF-SPME (CAR/PDMS and DVB/PDMS). In the last case, two different extraction procedures were tested: first, a simple extraction method, using only one kind of the TF-SPME and second, a dual extraction method using simultaneously the two kinds of TF-SPME (2TF-SPME: CAR/PDMS and DVB/PDMS). In all cases, 5 g EVOO was added into a special 20 mL headspace vial (Gerstel). The extractive device was then placed inside the vial in an open glass adapter (Gerstel). The vial was tightly capped for extraction. When the extraction device used was TF-SPME, the open glass insert was covered with a stainless steel adapter on which the TF-SPME was placed vertically in order to avoid contact between both devices in the dual extraction, in order to obtain a better extraction and prevent the fibre from falling into the sample.

The vial was then heated for 60 min at 37 °C in a thermostatic bath (BÜCHI Heating Bath B-490, New Castel, PA, USA). When the incubation time ended, the vial was kept at room temperature for five minutes and then the extractive device was removed with tweezers and, in the case of Twister^®^, these latter were rinsed with Milli-Q water and dried with lint-free tissue paper. The extractive device was transferred into a glass tube, 60 mm long, 6 mm o.d. and 4 mm i.d., which was then placed on the autosamples tray for thermal desorption in a gas chromatograph mass spectrometer (GC/MS). TF-SPME was handled using a special accessory provided by Gerstel, and a small piece of glass wool was placed into the desorption tube to prevent the fibre falling out of the tube. In the special case of the dual extraction, two TF-SPME had to be placed in the same desorption tube. In this situation, in order to achieve the correct simultaneous desorption, the CAR/PDMS thin film was put at the bottom of the desorption tube and the DVB/PDMS thin film was carefully placed above it.

### 2.4. Gas Chromatography-Mass Spectrometry (GC/MS) Analysis

Analyses were conducted using an Agilent 6890 GC system coupled to an Agilent 5975 inert quadrupole mass spectrometer (Agilent, Santa Clara, CA, US) equipped with a Gerstel Thermo Desorption System (TDS2) and a CIS-4 PTV inlet Cooling Injector System (Gerstel, Müllheim an der Ruhr, Germany). The desorption temperature program was as follows: the temperature was kept at 35 °C for 0.1 min, and then ramped at 60 °C/min to 220 °C and held for 5 min. The temperature of the CIS-4 PTV injector, with Tenax TA inlet liner, was held at −35 °C using liquid nitrogen for the total desorption time, and was then raised to 260 °C at a rate of 10 °C/s and held for 4 min. The solvent vent mode was used to transfer the sample to the analytical column. A 50 m × 0.25 mm J&W CPWax-57CB column and a film thickness of 0.20 µm (Agilent, Santa Clara, CA, USA) were used; the carrier gas was He at a 1 mL/min flow rate. The oven temperature program was as follows: 35 °C for 4 min and then raised to 220 °C at 2.5 °C/min (held for 15 min). The quadrupole, source and transfer line temperatures were maintained at 150 °C, 230 °C and 280 °C, respectively. The electron ionisation mass spectra were recorded in the full-scan mode at 70 eV with the electron energy in the range of 29 to 300 m/z.

### 2.5. Data Processing and Identification of Volatile Compounds

All data were processed using Deconvolution and Identification System (PARADISe) software. Chromatographic data were converted into netCDF format, exported to AIA format by MSD ChemStation (version F.01.01.2317). The first step was to define intervals using the total ion chromatogram, including one peak in each interval, making sure that this peak was completely inserted in the interval for the set chromatograms simultaneously processed. A total of 148 intervals were obtained (Table S1). For each interval, 8 compounds were then defined, including a baseline, enabling the PARAFAC2 model to solve the underlying and overlapping compounds in each interval [27]. In order to assess each model (i.e., the correct selection of number of components, Table S1), the fit and the core consistency were carefully optimised, attempting to reach values as close as possible to 100% for each parameter. The NIST MS Search program (version 2.0) was used for the preliminary identification of all components for each model, thus obtaining the optimal model. Finally, the PARADISe software created a report with the obtained data matrix that could be opened with Microsoft Excel, providing peak area values.

The NIST/EPA/NIH Mass Spectral Library was used for identification; the NIST MS Search program (v.2.0) assigned a volatile compound name to each deconvoluted mass spectrum profile obtained by the PARADISe software. Volatile compound identifications were confirmed based on comparisons of the linear retention index of standards (LRIs). LRIs were calculated using the retention times of a series of n-alkanes analysed under identical conditions to the samples.

### 2.6. Statistical Analysis

Significant differences among the data evaluation were performed by analysis of variance (ANOVA), followed by a *post hoc* comparison test (Tukey´s test) using INFOSTAT software (FCA, Universidad Nacional de Córdoba, Argentina), and principal component analysis (PCA) was undertaken using the PLS_Toolbox 7.9.5 (Eigenvector Research Inc., Wenatchee, WA, USA) working in a MATLAB environment. Data were autoescaled prior to PCA modelling

## 3. Results and Discussion

### 3.1. Evaluation of Different Extraction Methods to Determine Evoo Volatile Profiles

Several extraction devices, Twisters^®^ (PDMS and EG/S) and new TF-SPME (CAR/PDMS and DVB/PDMS), were compared in order to select the most suitable technique to characterise the volatile profiles of *Picual* and *Hojiblanca* variety EVOOs. A preliminary comparative approach was carried out: 11 volatile compounds, described by different authors [2,8,28] as active EVOO aromas, present in some of our samples were selected. The normalised peak area values of these compounds, obtained by manual integration, were normalised with respect to the mean values obtained using the HSSE-PDMS method (Table 1).

The HSSE-PDMS method provided higher values for the 11 active aromas selected than HSSE-EG/S for both varieties of EVOO, determining a lower number of these compounds in the *Picual* variety EVOO (Table 1). Similar results were found in sparkling wine by Ubeda et al. [24], who observed that, among other kinds of compounds, the PDMS polymeric phase turned out to be better than EG/S for extracting alcohols and aldehydes in headspace.

Comparing HSSE-PDMS with the new extraction devices, the worst results in the *Picual* variety EVOO were obtained with TF-SPME-CAR, since only 6 compounds were detected, and most of these presented the lowest values.

In the case of the *Hojiblanca* variety EVOO, there was no great difference between TF-SPME-CAR and HSSE-PDMS with respect to the number of compounds. However, the normalised peak area values were lower in the first case. Conversely, higher values in both EVOOs were observed for 1-peten-3-one and hexanal when TF-SPME-CAR was used. Many authors consider both volatile compounds to be characteristic of EVOO, with there being an active aroma in olive oil [2].

In the other extraction methods using TF-SPME-DVB and 2TF-SPME, we observed that 1-peten-3-one, hexanal and 2-methyl-2-butenal gave the highest normalised peak area values in both varieties of EVOO. Moreover, only these two TF-SPME methods (DVB/PDMS and 2TF) were able to determine the 2-methyl-2-butenal compound in our samples (Table 1).

As can be seen in Table 1, the highest normalised peak area values in the case of EVOO from the *Hojiblanca*-variety were obtained using HSSE-PDMS or 2TF-SPME, depending on the compound: 1-penten-3-ol for the first device or *cis*-3-hexenyl acetate for the second device, for example. However, in the case of *Picual* variety EVOO, the best devices turned out to be HSSE-PDMS and TF-SPME-DVB. In this case, the values obtained with TF-SPME-CAR and 2TF-SPME were very similar. In fact, there were no significant differences between them. Moreover, the relative standard deviation (RSD) values obtained using the CAR/PDMS extraction phase were higher than those using both extraction phases simultaneously (CAR/PDMS and DVB/PDMS) (data not shown).

Hence, the HSSE-PDMS and 2TF-SPME methods were selected for a more in-depth comparison.

### 3.2. Comparison of HSSE-PDMS and 2TF-SPME Methods

Since the HSSE-PDMS and 2TF-SPME methods provided suitable results for both kinds of EVOO, we focused on analysing which might be better for the proposed purpose.

In order to compare both sampling methods, three parameters usually used for analytical method validation were chosen: sensitivity, linearity and intermediate precision (inter-day precision). A refined olive oil (ROO) was spiked with different percentages of EVOO from the *Picual* variety (0, 25 and 50%). These mixtures and 100% EVOO were analysed in order to evaluate the sensitivity (extraction capacity) and linearity of these methods. In this comparative study, 13 compounds were considered, including relevant compounds in olive oil (detected by both methods in *Picual* EVOO) and attempting to ensure that all major chemical groups were represented, including at least two compounds from each chemical group. Results are shown in Table 2.

The sensitivity of the methods was evaluated considering the slope values of linear regression equations, built using the percentages of EVOO added to ROO as independent variables and peak area values as dependent variables (Table 2). With regard to the number of compounds, the results show that the sensitivity (extraction capacity) was similar. However, if we consider the chemical characteristics of these compounds, HSSE-PDMS shows better sensitivity values for alcohols, while 2TF-SPME does for ketones.

Twister^®^ PDMS extraction capacity for different volatile compounds depends on the polarity of these latter, and, moreover, its extraction capacity has been related to the octanol-water distribution coefficient (K_o/w_) of compounds [22]. In the case of alcohols, HSSE-PDMS extracted those whose log K_o/w_ values are higher than 0.9, the principle of differential extraction capacity as a function of polarity being fulfilled in this chemical group only [22].

In terms of linearity, correlation coefficients obtained for the 2TF-SPME ranged between 0.74 and 1.00 (Table 2), highlighting that 3-pentanone, *trans*-2-hexenal and *cis*-3-hexenol reached values of 1.00, whilst for HSSE-PDMS, these values ranged between 0.42 and 0.93. F-values of regression (Table 2) were significant for nine compounds in the case of the 2TF-SPME technique, but only for one compound, *cis*-3-hexenyl acetate, when we used the HSSE-PDMS method. In the case of the last compound, both techniques reached significant F-values, being better than the result in the case of 2-TF-SPME. Therefore, this sampling technique showed the best linearity.

In both techniques, intermediate precision, expressed as relative standard deviation (RSD), was calculated by analysing six replicates of the 100% EVOO over a period of 14 working days. RSD values were similar for both sampling methods (Table 2).

Both methods were very similar with regard to sensitivity and RSD values, and their primary difference was their linearity. Therefore, no clear conclusions regarding which was the best sampling method could be drawn. The next step to complete this comparison study was, therefore, to apply a non-targeting method, PARADISe software, to obtain more information from the sample chromatograms. Moreover, this was an opportunity to check the usefulness of this software when dealing with these types of samples.

### 3.3. Volatile Profile of EVOO by Non-Targeting Paradise Software

PARADISe software provided area values of 206 and 94 compounds for *Picual* and *Hojiblanca* variety EVOOs, respectively. In the *Picual* variety EVOO, 27 compounds were confirmed by standard mass spectrum and LRI; 11 were tentatively identified (TI) by mass spectrum, agreeing with the mass spectra from the NIST/EPA/NIH Mass Spectral Library data base, and LRI values with literature values. In addition, there were 15 with an unconfirmed identification, since only the compound mass spectrum matched with those from the NIST library (Table 3). In the case of the *Hojiblanca* variety EVOO, the identification of 17 compounds was confirmed; eight were TI and ten had unconfirmed identification (Table 3). Of the remaining compounds, some were contaminants from the analytical process. Others, reaching a low value of right identification probability for their mass spectrum in the library search, were considered unknown.

**Figure 1:**
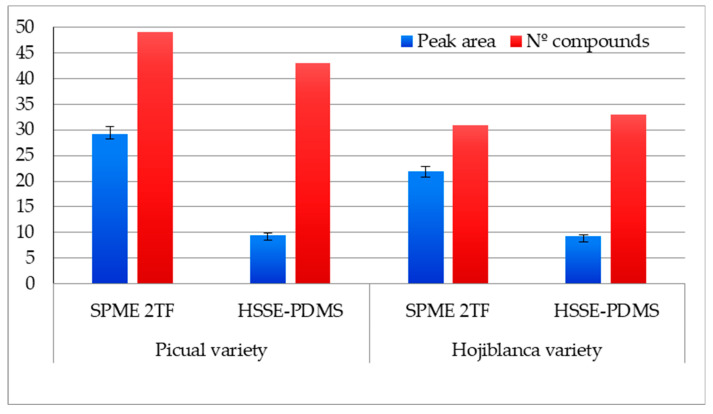
Values of total peak area (divided per 10^7^) and number of volatile compounds of EVOO *Picual* and *Hojiblanca* varieties obtained by HSSE-PDMS and 2TF-SPME. Error bars show standard deviation (SD) values.

In the *Picual* variety EVOO, a total of 49 and 43 compounds were determined with 2TF-SPME and HSSE-PDMS, respectively (Figure S1). In the *Hojiblanca* variety EVOO, HSSE-PDMS extracted a similar number of compounds (34) to 2TF-SPME (32) (Figure S2). However, the highest total area values were obtained using 2TF-SPME methods in both cases (Figure 1).

**Figure 2:**
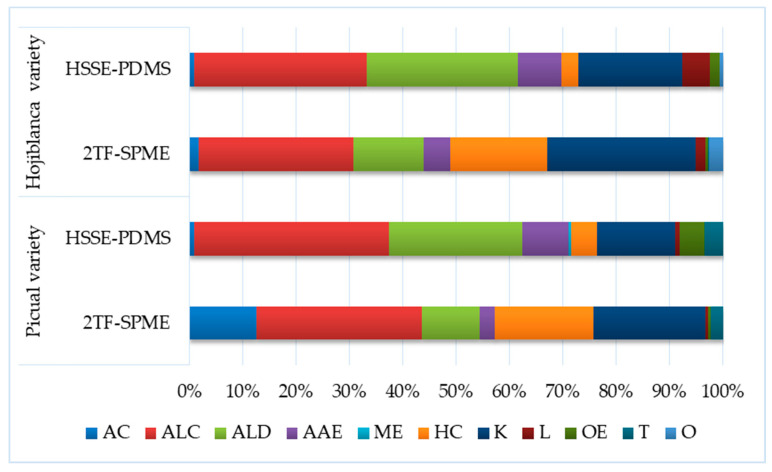
Percentage of total peak area values of major chemical groups of EVOO *Picual* and *Hojiblanca* varieties obtained by HSSE-PDMS and 2TF-SPME.

With regard to chemical groups, alcohols, aldehydes and ketones, based on their number of different compounds determined in the volatile profile of these olive oils, stood out. The total peak area values of each chemical group were statistically different for acids, alcohols, ketones and hydrocarbons, depending on the sampling methods used in both varieties (Table 3). Moreover, statistically significant differences were observed between sampling techniques for aldehydes and lactones for *Picual* olive oil and terpenes for *Hojiblanca*. In all cases, the highest values were reached using the 2TF-SPME method (Figure 2).

Independently of the EVOO variety, the 2TF-SPME device was the only method that detected the following eight volatile compounds: propanoic acid, 1-propanol, 2-methyl-2-pentenal, 5-hydroxymethylfurfural, 4-hexen-1-ol acetate, 2-cyclopentene-1, 3-dione and p-cymene, while *trans*-2-hexenol, *cis*-2-heptenal, heptadecane and n-hexyl salicylate were only detected using the PDMS Twister^®^ (Table 3). Several authors [29] who have studied odorant compounds in *Picual* olive oils from different countries and zones observed that *trans*-2-hexenol and *cis*-2-heptenal were of no importance in this variety.

When the 2TF-SPME method was used on the *Picual* variety sample, peak area values were significantly higher than with the HSSE-PDMS method for 23 volatile compounds, and for 14 volatile compounds, the values obtained were significantly higher in the case of the HSSE-PDMS sampling method (Table 3).

In the *Hojiblanca* variety sample, however, the highest number of compounds with the highest peak areas were obtained with the 2TF-SPME method; twelve volatile compounds as opposed to six volatile compounds when, in this variety, the extraction method was HSSE-PDMS.

Therefore, the results appear to show that the best sampling technique is 2TF-SPME.

**Figure 3:**
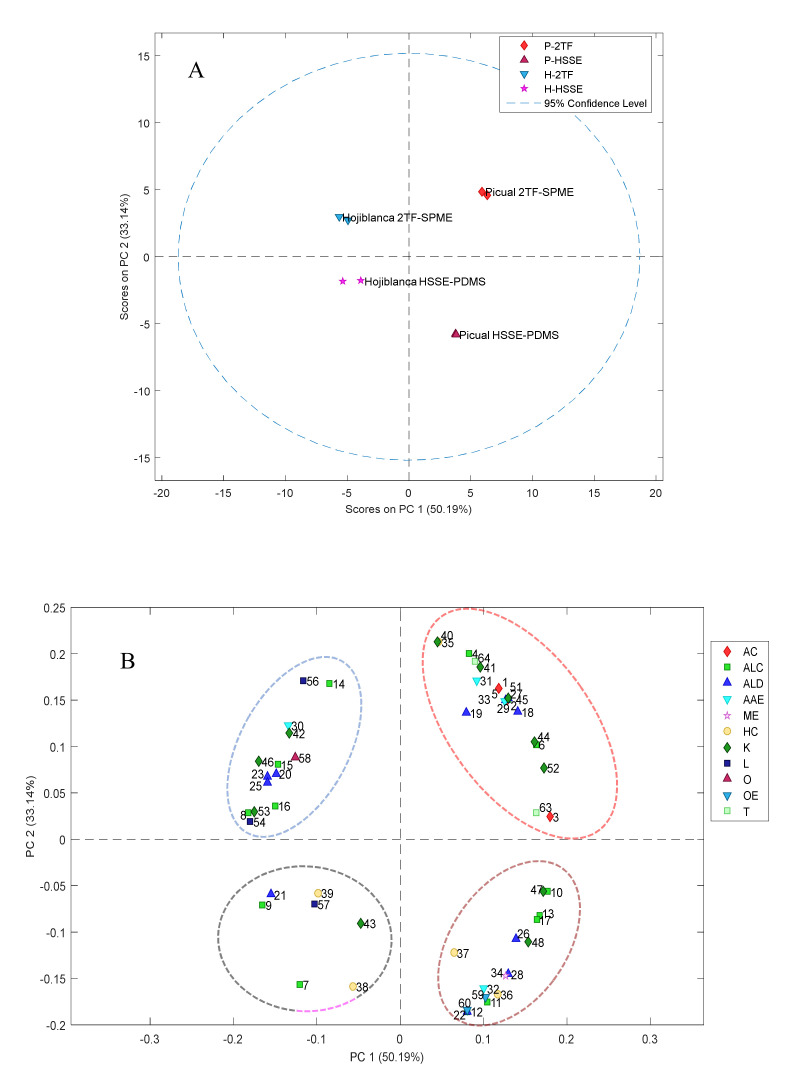
Application of PCA to the solved chromatographic profiles obtained by the HSSE-PDMS and 2TF-SPME extraction methods. (**A**) Score plot; (**B**) loading plot (the correspondence between numbers and volatile compounds is shown in Table 3).

Although few samples have been analysed in this work, a principal component analysis (PCA), as a tentative unsupervised statistical analysis, was performed. PC1, with a 50.19% explained variance, shows that EVOO samples could be separated according to the variety of oil, *Picual* or *Hojiblanca* (Figure 3), and PC2 with a 33.14% explained variance, could differentiate the samples studied according to the volatile compound extraction method used (Figure 3A). PCA confirmed the suitability of both techniques to differentiate *Picual* and *Hojiblanca* variety EVOOs. Regarding the loading values of variables, as can be seen in Figure 3B, a higher number of variables were correlated with samples analysed with 2TF-SPME than with HSSE-PDMS, supporting that the best sampling technique is 2TF-SPME. Although the total values of the area of several chemical groups showed significant differences between sampling methods, a clear correlation of compounds belonging to one chemical group with a sampling technique was not observed in PCA results, except for acids, aldehyde, ketones and hydrocarbons. All acids, most of aldehydes and the ketones seem to be correlated with the 2TF-SPME method, and hydrocarbons with the HSSE-PDMS sampling technique.

## 4. Conclusions

In this work, a comparative study to obtain the most suitable sampling method to determine the volatile profile of *Picual* and *Hojiblanca* variety EVOOs was performed. Among the five methods compared (HSSE-PDMS, HSSE-EG/S, TF-SPME-CAR, TF-SPME-DVB and 2TF-SPME), the best results were achieved by HSSE-PDMS and 2TF-SPME. Both extraction methods enable EVOO samples to be separated and differentiated according to the olive varieties. However, taking into account the linearity and the peak area values obtained, as well as the number of volatile compounds determined, the 2TF-SPME method turned out more suitable to best characterize these types of EVOOs.

This study is a first approach to the use of the new TF-SPME sampling method to determine the volatile profile of EVOOs. Therefore, further research including a high number of EVOOs from different olive oil varieties is needed to confirm and guarantee its suitability for the characterization and differentiation of a wide range of EVOO samples.

Nevertheless, the new device, TF-SPME, currently has a high price and, possibly, a shorter shelf life than the PDMS Twister^®^ device (ten times lower), as pointed out by the supplier. These facts could mean that the HSSE-PDMS method continues to be used as a sampling method to determine the volatile profile of EVOO, instead of the new TF-SPME device.

## Figures and Tables

**Table 1:** Comparison of volatile compounds by different extraction methods. Peak relative area normalized with respect to HSSE-PDMS.

Volatile Compound	*Picual* Variety EVOO					*Hojiblanca* Variety EVOO				
	HSSE-PDMS	HSSE-EG/S	TF-SPME-CAR	TF-SPME-DVB	2TF-SPME	HSSE-PDMS	HSSE-EG/S	TF-SPME-CAR	TF-SPME-DVB	2TF-SPME
1-Penten-3-one	1	nq ^a^	2.11 ^b^	3.31 ^c^	3.26 ^c^	1	0.23 ^a^	2.62 ^b^	2.15 ^b^	2.79 ^b^
Hexanal	1	0.18 ^a^	1.07 ^b^	1.40 ^c^	1.10 ^b,c^	1	0.53 ^a^	1.41 ^b^	1.33 ^b^	1.49 ^b^
2-Methyl-2-butenal	nq	nq	nq	1	0.68	nq	nq	nq	1	1.11
*cis*-3-Hexenal	nq	nq	np	nq	nq	1	nq	nq	nq	nq
1-Penten-3-ol	1	0.48 ^a^	0.21 ^a^	0.58 ^a^	0.18 ^a^	1	0.89 ^a^	0.38 ^b^	0.19 ^b^	0.36 ^b^
*trans*-2-Hexenal	1	nq ^a^	0.53 ^b^	1.01 ^c^	0.72 ^b,c^	1	0.57 ^a^	0.67 ^a^	0.74 ^a^	0.71 ^a^
*cis*-3-Hexenyl acetate	1	0.54 ^a^	0.49 ^a^	0.88 ^b^	0.72 ^a,b^	1	0.80 ^a^	0.88 ^a, b^	0.74 ^a^	1.07 ^b^
*cis*-2-Penten-1-ol	1	0.67 ^a^	nq ^a^	nq ^a^	nq ^a^	1	0.98 ^a^	0.29 ^b, c^	nq ^c^	0.42 ^b^
1-Hexanol	1	0.77 ^a^	nq ^b^	1.02 ^a^	0.84 ^a^	1	0.89 ^a, b^	0.74 ^a, b^	0.73 ^a^	1.03 ^b^
*cis*-3-Hexenol	1	0.73 ^a^	0.30 ^b^	0.67 ^a^	0.45 ^a, b^	1	0.99 ^a^	0.60 ^a, b^	0.42 ^b^	0.84 ^a,b^
*trans*, *trans*-2,4-Heptadienal	1	0.53 ^a^	nq ^a^	0.57 ^a^	0.36 ^a^	nq	nq	nq	nq	nq

Underlined values correspond to the highest values found. Similar superscript letters in the same row indicate no statistically significant differences (*p* < 0.05) for each variety of EVOO. nq: lower than quantification limit (a signal-to-noise ratio >10), np: peak not detected or lower than detection limit (a signal-to-noise ratio >3).

**Table 2:** Results of sensitivity, linearity and intermediate precision studies.

No	Compound	Log K_o/w_^a^	LRIexp ^b^	Qualifier Ion ^c^	Linear Regression Equation		r^2^		F-Value		RSD	
					PDMS	2TF	PDMS	2TF	PDMS	2TF	PDMS	2TF
1	Propanal	0.59	825	58	y = 730,789x + 210,565	y = 8,000,000x + 2,000,000	0.78	0.74	7.1	5.7	10	0
2	3-Pentanone	0.99	945	86	y = 5,000,000x + 324,716	y = 10,000,000x + 2,000,000	0.88	1.00	15.3	502.5 *	2	3
3	1-Penten-3-one	1.15	987	55	y = 1,000,000x + 90,747	y = 4,000,000x + 281,472	0.83	0.99	9.9	202.2 *	5	8
4	Hexanal	1.78	1047	44	y = 9,000,000x + 3,000,000	y = 10,000,000x + 3,000,000	0.89	0.99	15.6	166.6 *	7	1
5	1-Butanol	0.88	1136	41	y = 132,370x + 68,115	y = 490,345x + 193,511	0.67	0.98	3.9	86.4 *	8	2
6	1-Penten-3-ol	0.94	1148	57	y = 3,000,000x + 800,614	y = 430,521x + 484,730	0.78	0.80	7.1	7.8	1	6
7	*trans*-2-Hexenal	1.58	1198	41	y = 9,000,000x + 1,000,000	y = 6,000,000x + 722,811	0.90	1.00	17.7	1553.6 *	7	6
8	Hexyl acetate	2.13	1251	105	y = 217,332x – 20,613	y = 345,736x + 93,668	0.55	0.90	2.4	17.4	3	5
9	*cis*-3-Hexenyl acetate	-	1293	43	y = 7,000,000x + 641,679	y = 4,000,000x + 778,562	0.93	0.99	27.5 *	281.6 *	8	4
10	1-Hexanol	2.03	1344	56	y = 1,000,000x + 192,735	y = 757,743x + 259,098	0.78	0.96	7.0	49.3 *	2	4
11	1-Hydroxy-2-butanone	−0.04	1356	88	y = 259,719x + 241,191	y = 166,645x + 300,871	0.42	0.86	1.4	11.9	1	13
12	*cis*-3-Hexenol	1.61	1371	67	y = 10,000,000x + 2,000,000	y = 4,000,000x + 1,000,000	0.86	1.00	12.5	440.6 *	10	7
13	*trans, trans*-2,4-Heptadienal	-	1443	81	y = 1,000,000x + 197,269	y = 448,741x + 6855.7	0.73	0.98	5.4	78.8 *	9	13

^a^ Logarithm of octanol/water partition coefficient. -: the Log K _o/w_ values were not found for these volatile compounds. ^b^ LRIexp: Experimetal Linear Retention Index. * = F-values significant. ^c^ m/z data.

**Table 3:** Volatile compounds determined in *Picual* and *Hojiblanca* varieties of EVOO by different sampling methods.

Nº	Volatile Compounds	LRIexp	ID	Chemical Group	Peak Area											
					*Picual* Variety EVOO						*Hojiblanca* Variety EVOO					
					2TF-SPME			HSSE-PDMS			2TF-SPME			HSSE-PDMS		
					Am	SD		Am	SD		Am	SD		Am	SD	
	**Acids**															
1	Aceticacid	1435	A	AC	31,118,420	7,445,518	^b^	516,280	139,167	^a^	3,837,461	261,442	^d^	882,064	223,544	^c^
2	Propanoicacid	1527	A	AC	5,004,955	741,422	^b^	nq	‒	^a^	np	‒		np	‒	
3	4-Hydroxybutanoicacid	1617	C	AC	566,580	139,245	^a^	389,572	27,499	^a^	np	‒		np	‒	
	**Total of Acids**				**36,689,955**	**8,326,185**	**^b^**	**905,851**	**166,666**	**^a^**	**3,837,461**	**261,442**	**^d^**	**882,064**	**223,544**	**^c^**
	Alcohols															
4	Ethanol	918	A	ALC	78,415,584	6,936,582	^b^	12,513,436	1,536,408	^a^	43,560,290	8,601,346	^d^	9,390,870	500,465	^c^
5	1-Propanol	1022	A	ALC	583,812	68,905	^b^	nq	‒	^a^	np	‒		np	‒	
6	1-Butanol	1136	A	ALC	594,886	6595	^b^	177,248	16,643	^a^	np	‒		np	‒	
7	1-Penten-3-ol	1146	A	ALC	286,561	1392	^a^	2,701,691	49,259	^b^	2,085,424	120,909	^c^	4,243,629	84,472	^d^
8	2,4-Hexadien-1-ol	1180	C	ALC	np	‒		np	‒		4,328,335	49,850	^c^	4,332,935	284,485	^c^
9	*cis-*2-Penten-1-ol	1311	A	ALC	394,825	7575	^a^	1,447,307	71,582	^b^	2,431,735	219,582	^c^	3,773,490	324,856	^d^
10	1-Hexanol	1343	A	ALC	1,106,427	13,981	^a^	1,339,551	38,490	^b^	np	‒		np	‒	
11	*cis*-3-Hexen-1-ol	1372	A	ALC	3,991,048	408,946	^a^	11,200,860	1,142,815	^b^	2,141,330	401,895	^c^	3,139,178	494,894	^c^
12	*trans*-2-Hexenol	1392	A	ALC	nq	‒	^a^	588,914	8970	^b^	np	‒		np	‒	
13	2-Ethyl-1-hexanol	1479	A	ALC	453,162	21,880	^a^	670,229	23,234	^b^	np	‒		np	‒	
14	2-Furanmethanol	1653	A	ALC	2,032,397	21,240	^b^	1,251,341	94,612	^a^	2,831,237	36,724	^c^	1,644,682	582,425	^c^
15	1-Dodecanol	1969	A	ALC	1,396,587	212,229	^a^	1,364,093	180,607	^a^	4,392,903	110,859	^d^	2,219,065	662,197	^c^
16	1-Tetradecanol	2177	B ^1^	ALC	677,716	92,869	^a^	815,976	63,503	^a^	1,597,754	244,979	^c^	1,112,394	524,545	^c^
17	1-Hexadecanol	2379	B ^2^	ALC	471,960	23,786	^a^	724,227	119,290	^a^	np	‒		np	‒	
	**Total of Alcohols**				**90,404,966**	**7,845,980**	**^b^**	**34,794,869**	**3,345,413**	**^a^**	**63,369,008**	**9,786,145**	**^d^**	**29,856,241**	**3,458,339**	**^c^**
	**Aldehydes**															
18	Propanal	825	C	ALD	10,499,980	27,176	^b^	1,032,601	89,641	^a^	np	‒		np	‒	
19	Hexanal	1047	A	ALD	9,254,006	139,578	^b^	7,691,922	434,528	^a^	8,934,458	378,583	^d^	5,338,161	272,680	^c^
20	*trans*-2-Pentenal	1096	B ^3^	ALD	2,520,827	24,806	^a^	2,699,071	33,358	^b^	5,818,509	53,918	^d^	3,478,926	139,944	^c^
21	2-Hexenal	1197	B ^3^	ALD	6,448,170	507,767	^a^	8,240,025	982,418	^a^	10,541,677	1,146,435	^c^	14,632,251	1,364,275	^c^
22	*cis*-2-Heptenal	1302	C	ALD	nq	‒	^a^	421,612	2145	^b^	np	‒		np	‒	
23	*trans,trans*-2,4-Hexadienal	1379	A	ALD	np	‒		np	‒		1,001,242	367,404	^c^	450,643	46,512	^c^
24	3-Furaldehyde	1400	C	ALD	nq	‒		nq	‒		np	‒		np	‒	
25	2-Furfuraldehyde	1438	A	ALD	1,429,274	474,509	^a^	1,349,695	444,360	^a^	2,563,006	244,705	^c^	2,134,954	195,650	^c^
26	5-Methylfurfural	1554	B ^4^	ALD	220,396	9543	^a^	268,105	77,534	^a^	nq	‒	^c^	168,936	5133	^d^
27	2-Methyl-2-pentenal	1710	C	ALD	196,730	44,226	^b^	nq	‒	^a^	np	‒		np	‒	
28	α-Hexylcinnamicaldehyde	2358	C	ALD	259,344	45,673	^a^	813,664	46,349	^b^	np	‒		np	‒	
29	5-Hydroxymethylfurfural	2476	A	ALD	164,593	477	^b^	nq	‒	^a^	np	‒		np	‒	
	**Total of Aldehydes**				**30,993,319**	**1,273,755**	**^b^**	**22,516,695**	**2,110,334**	**^a^**	**28,858,892**	**2,191,044**	**^c^**	**26,203,870**	**2,024,195**	**^c^**
	**Acetic Acid Esters**															
30	Methylacetate	838	A	AAE	1,584,797	793,118	^a^	281,374	2684	^a^	5,885,996	71,117	^d^	2,215,590	565,841	^c^
31	Hexylacetate	1251	A	AAE	817,794	89,701	^b^	235,110	17,904	^a^	346,876	28,628	^c^	374,931	104,272	^c^
32	*cis*-3-Hexenylacetate	1293	A	AAE	5,442,934	35,393	^a^	7,639,594	15,574	^b^	5,008,824	261,508	^c^	4,983,399	803,358	^c^
33	4-Hexen-1-olacetate	1308	C	AAE	404,617	114,097	^b^	nq	‒	^a^	np	‒		np	‒	
	**Total of Acetic Acid Esters**				**8,250,142**	**1,032,308**	**^a^**	**8,156,078**	**36,163**	**^a^**	**11,241,697**	**361,253**	**^c^**	**7,573,920**	**1,473,471**	**^c^**
	**Methyl Ester**															
34	Methylpalmitate	2205	B ^3^	ME	167,003	17,989	^a^	552,486	61,210	^b^	np	‒		np	‒	
	**Total of Methyl Ester**				**167,003**	**17,989**	**^a^**	**552,486**	**61,210**	**^b^**	‒	‒		‒	‒	
	**Hydrocarbons**															
35	Pentane	789 *	C	HC	51,900,552	2,656,368	^b^	nq	‒	^a^	38,214,752	6,226,335	^d^	np	‒	^c^
36	1-Tridecene	1223	C	HC	1,759,219	49,467	^a^	4,063,632	88,718	^b^	800,849	8859	^c^	1,254,160	321,816	^c^
37	Hexadecane	1582	C	HC	205,693	7464	^a^	258,029	97,175	^a^	nq	‒	^c^	304,889	31,778	^d^
38	Heptadecane	1685	C	HC	nq	‒	^a^	315,619	115,316	^a^	nq	‒	^c^	608,734	35,513	^d^
39	Octadecane	1786	C	HC	np	‒		np	‒		nq	‒	^c^	533,427	81,897	^d^
	**Total of Hydrocarbons**				**53,865,464**	**2,713,299**	**^b^**	**4,637,280**	**301,209**	**^a^**	**39,015,601**	**6,235,195**	**^d^**	**2,701,210**	**471,003**	**^c^**
	**Ketones**															
40	Acetone	834	A	K	30,677,216	4,809,497	^b^	np	‒	^a^	21,413,297	5,732,598	^d^	1,323,780	127,696	^c^
41	3-Pentanone	945	C	K	17,626,991	237,216	^b^	5,536,161	132,675	^a^	10,814,458	1,196,099	^d^	2,116,622	196,558	^c^
42	1-Penten-3-one	987	B ^3^	K	4,226,219	355,741	^b^	704,886	20,647	^a^	21,836,991	3,208,204	^d^	6,417,422	92,595	^c^
43	1-Hydroxy-2-propanone	1285	A	K	4,869,773	494,071	^a^	5,810,049	42,804	^a^	5,009,257	256,047	^c^	6,512,853	2,871,872	^c^
44	6-Methyl-5-hepten-2-one	1316	A	K	797,763	15,837	^b^	223,814	759	^a^	np	‒		np	‒	
45	2-Cyclopenten-1-one	1341	B ^5^	K	353,731	15,462	^b^	nq	‒	^a^	np	‒		np	‒	
46	1-Hydroxy-2-butanone	1356	B ^3^	K	968,193	58,337	^b^	601,323	35,573	^a^	1,929,058	19,807	^c^	1,784,378	269,315	^c^
47	2-Acetylfuran	1484	A	K	234,449	18,167	^a^	286,228	78,029	^a^	np	‒		np	‒	
48	*(trans,trans)*-3,5-Octadien-2-one	1502	C	K	167,456	2333	^a^	320,586	11,295	^b^	np	‒		np	‒	
49	3-Methyl-2-cyclopenten-1-one	1513	B ^3^	K	nq	‒		nq	‒		np	‒		np	‒	
50	3,5-Octadien-2-one	1556	C	K	nq	‒		nq	‒		np	‒		np	‒	
51	4-Cyclopentene-1,3-dione	1561	C	K	182,399	10,564	^b^	nq	‒	^a^	np	‒		np	‒	
52	Acetophenone	1632	A	K	928,088	55,308	^b^	391,497	31,874	^a^	np	‒		np	‒	
53	5-Hydroxymethyldihydrofuran-2-one	2511	C	K	np	‒		np	‒		384,452	76,782	^c^	372,613	193,649	^c^
	**Total of Ketones**				**61,032,278**	**6,072,532**	**^b^**	**13,874,544**	**353,656**	**^a^**	**61,387,512**	**10,489,537**	**^d^**	**18,527,669**	**3,751,685**	**^c^**
	**Lactones**															
54	γ-Butyrolactone	1617	A	L	np	‒		np	‒		1,782,013	158,906	^c^	2,076,950	427,063	^c^
55	5-Methyl-2(5H)-furanone	1668	B ^3^	L	nq	‒		nq	‒		np	‒		np	‒	
56	2(5H)-Furanone	1747	B ^3^	L	1,099,855	98,614	^b^	309,921	49,402	^a^	1,532,727	151,332	^c^	1,173,518	287,649	^c^
57	2-Hydroxy-γ-butyrolactone	2191	C	L	391,104	42,638	^a^	591,424	94,875	^a^	607,702	172,744	^c^	1,080,110	670,105	^c^
	**Total of Lactones**				**1,490,959**	**141,251**	**^b^**	**901,345**	**144,278**	**^a^**	**3,922,441**	**482,982**	**^c^**	**4,330,578**	**1,384,817**	**^c^**
	**Other**															
58	*cis*-1-Methoxy-3-hexene	976	B ^3^	O	np	‒		np	‒		5,635,249	210,581	^d^	619,044	468,416	^c^
	**Total of Other**				‒	‒		‒	‒		**5,635,249**	**210,581**	**^d^**	**619,044**	**468,416**	**^c^**
	**Other Esters**															
59	2,2,4-Trimethyl-1,3-pentanedioldiisobutyrate	1871	C	OE	1,843,781	299,958	^a^	4,313,241	986,633	^a^	838,526	184,983	^c^	1,638,700	697,523	^c^
60	n-Hexylsalicylate	2202	B ^6^	OE	nq	‒	^a^	170,168	27,757	^b^	np	‒		np	‒	
	**Total of Other Esters**				**1,843,781**	**299,958**	**^a^**	**4,483,409**	**1,014,390**	**^a^**	**838,526**	**184,983**	**^c^**	**1,638,700**	**697,523**	**^c^**
	**Volatile Phenol**															
61	Guaiacol	1855	A	VP	nq	‒		nq	‒		np	‒		np	‒	
	**Total of Volatile Phenol**				‒	‒		‒	‒		‒	‒		‒	‒	
	**Pyrazine**															
62	Methylpyrazine	1255	B ^3^	PYR	nq	‒		nq	‒		np	‒		np	‒	
	**Total of Pyrazine**				‒	‒		‒	‒		‒	‒		‒	‒	
	**Terpenes**															
63	Limonene	1161	A	T	5,052,156	3,131,350	^a^	3,201,960	573,678	^a^	np	‒		np	‒	
64	p-Cymene	1240	B ^3^	T	1,389,156	392,534	^b^	nq	‒	^a^	508,539	66,747	^d^	nq	‒	^c^
	**Total of Terpenes**				**6,441,312**	**3,523,884**	**^a^**	**3,201,960**	**573,678**	**^a^**	**508,539**	**66,747**	**^d^**	‒	‒	**^c^**

LRIexp: Experimetal Linear Retention Index.; * LRI values estimated by linear regression. ID: reliability of identification: A, mass spectrum and LRI agreed with standards; B, mass spectrum agreed with mass spectral data base and LRI agreed with the literature data; C, mass spectrum agreed with mass spectral data base; ^a^ Literature reference agreed with LRI data: ^1^. Choi, Kim, & Sawamura (2002); ^2^. Liang, Chen, Reeves, & Han, (2013); ^3^. National Center for Biotechnology Information (2004); ^4^. Fan, & Qian (2006); ^5^. Chevance, & Farmer (1999); ^6^. Lukić, Radeka, Grozaj, Staver, & Peršurić (2016). References list provided in Supplementary Material. Chemical group: AC, acid; ALC, alcohol; ALD, aldehyde; AAE, acetic acid ester; EM, methyl ester; HC, hydrocarbures; K, ketones; L, lactones; O, other; OE, other ester; PV, volatile phenol; PYR, pyrazine; T, terpene. Am: mean area values; SD: standard deviation; nq: lower than quantification limit (a signal-to-noise ratio >10), np: peak not detected or lower than detection limit (a signal-to-noise ratio >3). Similar superscript letters in the same row indicate no statistically significant differences (*p* < 0.05) for each variety of EVOO; ^a,b^ for the *Picual* variety and ^c,d^ for the *Hojiblanca* variety EVOO.

## Supplementary Materials

The following are available online at https://www.mdpi.com/2304-8158/9/6/748/s1, Figure S1: Overlapping chromatograms of *Hojiblanca* variety EVOO obtained by 2TF-SPME and HSSE-PDMS methods., Figure S2: Overlapping chromatograms of *Picual* variety EVOO obtained by 2TF-SPME and HSSE-PDMS methods. Table S1: Intervals and number of components in the data processing by PARADISe.

## References

[1] Cecchi T., Alfei B. (2013). Volatile profiles of Italian monovarietal extra virgin olive oils via HS-SPME-GC-MS: Newly identified compounds, flavors molecular markers, and terpenic profile. Food Chem..

[2] Luna G., Morales M.T., Aparicio R. (2006). Characterisation of 39 varietal virgin olive oils by their volatile compositions. Food Chem..

[3] Vichi S., Castellote A.I., Pizzale L., Conte L.S., Buxaderas S., López-Tamames E. (2003). Analysis of virgin olive oil volatile compounds by headspace solid-phase microextraction coupled to gas chromatography with mass spectrometric and flame ionization detection. J. Chromatogr. A.

[4] International Olive Council COI/T.20/Doc. No 15/Rev. 10. https://www.internationaloliveoil.org/what-we-do/chemistry-standardisation-unit/standards-and-methods/.

[5] Guclu G., Sevindik O., Kelebek H., Selli S. (2016). Determination of Volatiles by Odor Activity Value and Phenolics of cv. Ayvalik Early-Harvest Olive Oil. Foods.

[6] Ministerio de Agricultura, Pesca y Alimentación. https://www.mapa.gob.es/es/agricultura/temas/producciones-agricolas/aceite-oliva-y-aceituna-mesa/aceite.aspx.

[7] Vichi S. (2010). Extraction Techniques for the Analysis of Virgin Olive Oil Aroma.

[8] Chtourou F., Ben Brahim S., Bouaziz M., Selli S., Amanpour A., Kelebek H. (2018). Gas Chromatography–Mass Spectrometry–Olfactometry To Control the Aroma Fingerprint of Extra Virgin Olive Oil from Three Tunisian Cultivars at Three Harvest Times. J. Agric. Food Chem..

[9] Perestrelo R., Silva C., Silva P., Câmara J.S. (2017). Global volatile profile of virgin olive oils flavoured by aromatic/medicinal plants. Food Chem..

[10] Barbieri S., Brkic Bubola K., Bendini A., Bucar-Miklavcic M., Lacoste F., Tibet U., Winkelmann O., García-González D.L., Toschi T.G. (2020). Alignment and Proficiency of Virgin Olive Oil Sensory Panels: The OLEUM Approach. Foods.

[11] Kanavouras A., Kiritsakis A., Hernandez R.J. (2005). Comparative study on volatile analysis of extra virgin olive oil by dynamic headspace and solid phase micro-extraction. Food Chem..

[12] Cavalli J.F., Fernandez X., Lizzani-Cuvelier L., Loiseau A.M. (2003). Comparison of Static Headspace, Headspace Solid Phase Microextraction, Headspace Sorptive Extraction, and Direct Thermal Desorption Techniques on Chemical Composition of French Olive Oils. J. Agric. Food Chem..

[13] Vichi S., Guadayol J.M., Caixach J., López-Tamames E., Buxaderas S. (2007). Comparative study of different extraction techniques for the analysis of virgin olive oil aroma. Food Chem..

[14] Quintanilla-Casas B., Bustamante J., Guardiola F., García-González D.L., Barbieri S., Bendini A., Toschi T.G., Vichi S., Tresa A. (2020). Virgin olive oil volatile fingerprint and chemometrics: Towards an instrumental screening tool to grade the sensory quality. LWT-Food Sci. Technol..

[15] Oliver-Pozo C., Aparicio-Ruiz R., Romero I., García-González D.L. (2015). Analysis of Volatile Markers for Virgin Olive Oil Aroma Defects by SPME-GC/FID: Possible Sources of Incorrect Data. J. Agric. Food Chem..

[16] Ríos-Reina R., Morales M., García-González D.L., Amigo J.M., Callejón R.M. (2018). Sampling methods for the study of volatile profile of PDO wine vinegars. A comparison using multivariate data analysis. Food Res. Int..

[17] Westland J., Technologies A. Sample Preparation SPME Arrow Sampling of Terpenes in Cannabis Plant Material. https://www.agilent.com/cs/library/applications/application-spme-arrow-sampling-of-terpenes-in-cannabis-plant-material-5994-1046en-agilent.pdf.

[18] Boyaci E., Goryński K., Viteri C.R., Pawliszyn J. (2016). A study of thin film solid phase microextraction methods for analysis of fluorinated benzoic acids in seawater. J. Chromatogr. A.

[19] Marsili R.T., Laskonis C.R. (2019). Evaluation of Sequential-SBSE and TF-SPME Extraction Techniques Prior to GC-TOFMS for the Analysis of Flavor Volatiles in Beer. J. Am. Soc. Brew. Chem..

[20] Song N.E., Lee J.Y., Lee Y.Y., Park J.D., Jang H.W. (2019). Comparison of headspace–SPME and SPME-Arrow–GC–MS methods for the determination of volatile compounds in Korean salt–fermented fish sauce. Appl. Biol. Chem..

[21] Stuff J.R., Whitecavage J.A., Grandy J.J., Pawliszyn J. (2018). Analysis of Beverage Samples using Thin Film Solid Phase Microextraction (TF-SPME) and Thermal Desorption GC/MS. GERSTEL App. Note.

[22] David F., Sandra P. (2007). Stir bar sorptive extraction for trace analysis. J. Chromatogr. A.

[23] Hoffmann A., Sandra P., David F. (2000). A novel extraction technique for aqueous samples: Stir bar sorptive extraction. Gerstel.

[24] Ubeda C., Callejón R.M., Troncoso A.M., Peña-Neira A., Morales M.L. (2016). Volatile profile characterisation of Chilean sparkling wines produced by traditional and Charmat methods via sequential stir bar sorptive extraction. Food Chem..

[25] Callejón R.M., González A.G., Troncoso A.M., Morales M.L. (2008). Optimization and validation of headspace sorptive extraction for the analysis of volatile compounds in wine vinegars. J. Chromatogr. A.

[26] Bicchi C., Cordero C., Iori C., Rubiolo P., Sandra P. (2000). Headspace Sorptive Extraction (HSSE) in the Headspace Analysis of Aromatic and Medicinal Plants. J. High Resolut. Chromatogr..

[27] Johnsen L.G., Skou P.B., Khakimov B., Bro R. (2017). Gas chromatography–mass spectrometry data processing made easy. J. Chromatogr. A.

[28] Peres F., Jeleń H.H., Majcher M.M., Arraias M., Martins L.L., Ferreira-Dias S. (2013). Characterization of aroma compounds in Portuguese extra virgin olive oils from Galega Vulgar and Cobrançosa cultivars using GC-O and GC×GC-ToFMS. Food Res. Int..

[29] Genovese A., Caporaso N., Leone T., Paduano A., Mena C., Perez-Jimenez M.A., Sacchi R. (2019). Use of odorant series for extra virgin olive oil aroma characterisation. J. Sci. Food Agric..

