# Supplementary material for: Comparison of the Novel Thin Film-Solid Phase Microextraction and Sorptive Extraction Methods for Picual and Hojiblanca Olive Oil Volatile Fraction Analysis in Headspace
Source: Foods. 2020 Jun 5;9(6):748. doi: 10.3390/foods9060748 (PMC7353552; doi:10.3390/foods9060748)
Supplement: Supplementary file 1 [file foods-09-00748-s001.doc]

Table S1. Intervals an number of components chosen in the data processing by PARADISe.

| **Intervals** | **Hojiblanca EVOO** | | | **Picual EVOO** | | |
| --- | --- | --- | --- | --- | --- | --- |
|  | **Start** | **End** | **Nº of components** | **Start** | **End** | **Nº of components** |
| 1 | 2,2809 | 2,8733 | 1 | 2,2808 | 2,8732 | 2 |
| 2 | 3,8552 | 4,6210 | 2 | 3,8551 | 4,6209 | 2 |
| 3 | 4,6243 | 5,0269 | 3 | 4,6242 | 5,0268 | 1 |
| 4 | 5,0334 | 5,3705 | 0 | 5,0333 | 5,3704 | 1 |
| 5 | 5,3771 | 5,7175 | 0 | 5,3770 | 5,7174 | 2 |
| 6 | 5,7338 | 6,3230 | 2 | 5,7337 | 6,3229 | 4 |
| 7 | 6,5913 | 6,9743 | 3 | 6,5912 | 6,9742 | 3 |
| 8 | 6,5913 | 7,3310 | 0 | 7,0003 | 7,3309 | 1 |
| 9 | 7,0005 | 7,6681 | 2 | 7,3604 | 7,6680 | 0 |
| 10 | 7,3605 | 8,0478 | 2 | 7,7040 | 8,0477 | 3 |
| 11 | 7,7410 | 8,4732 | 0 | 8,1884 | 8,4732 | 3 |
| 12 | 8,1885 | 9,2915 | 1 | 8,4862 | 9,2914 | 2 |
| 13 | 8,4864 | 9,9199 | 3 | 9,5205 | 9,9198 | 3 |
| 14 | 9,5206 | 10,4501 | 1 | 9,9558 | 10,4500 | 2 |
| 15 | 9,9559 | 10,9410 | 3 | 10,6005 | 10,9409 | 2 |
| 16 | 10,6006 | 11,2781 | 3 | 11,0096 | 11,2780 | 2 |
| 17 | 11,0098 | 11,5072 | 0 | 11,2977 | 11,4071 | 2 |
| 18 | 11,2978 | 12,4302 | 2 | 11,5922 | 12,4301 | 3 |
| 19 | 11,5923 | 12,7444 | 2 | 12,4530 | 12,7443 | 3 |
| 20 | 12,4531 | 14,0307 | 2 | 13,4250 | 14,0306 | 3 |
| 21 | 13,4251 | 15,4347 | 0 | 15,1368 | 15,4346 | 2 |
| 22 | 15,1369 | 15,7849 | 3 | 15,5426 | 15,7848 | 2 |
| 23 | 15,5427 | 16,2333 | 2 | 15,8732 | 16,2332 | 3 |
| 24 | 15,8733 | 17,0941 | 3 | 16,8354 | 17,0940 | 2 |
| 25 | 16,8355 | 18,0203 | 0 | 17,6864 | 18,0202 | 3 |
| 26 | 17,6865 | 18,6291 | 1 | 18,0464 | 18,6290 | 3 |
| 27 | 18,0465 | 19,1429 | 1 | 18,6355 | 19,1428 | 2 |
| 28 | 18,6356 | 19,9677 | 1 | 19,3032 | 19,9676 | 3 |
| 29 | 19,3033 | 20,3015 | 0 | 20,0363 | 20,3014 | 3 |
| 30 | 20,0364 | 20,4292 | 0 | 20,3211 | 20,4291 | 2 |
| 31 | 20,3212 | 21,1263 | 2 | 20,4422 | 21,1262 | 2 |
| 32 | 20,4423 | 22,3995 | 3 | 21,5713 | 22,3994 | 3 |
| 33 | 21,2714 | 22,9133 | 2 | 22,6121 | 22,9132 | 2 |
| 34 | 22,6122 | 23,2111 | 2 | 22,9361 | 23,2111 | 2 |
| 35 | 22,9362 | 23,5221 | 3 | 23,2700 | 23,5220 | 2 |
| 36 | 23,2701 | 23,7348 | 2 | 23,5645 | 23,7347 | 3 |
| 37 | 23,5646 | 24,5072 | 0 | 23,9147 | 24,5071 | 3 |
| 38 | 23,9148 | 25,2992 | 3 | 24,8671 | 25,2992 | 2 |
| 39 | 24,8672 | 25,9080 | 1 | 25,5086 | 25,9079 | 3 |
| 40 | 25,5087 | 26,2779 | 2 | 26,0192 | 26,2778 | 3 |
| 41 | 26,0193 | 26,9164 | 1 | 26,3563 | 26,9160 | 4 |
| 42 | 26,3564 | 28,5460 | 0 | 27,8749 | 28,5459 | 4 |
| 43 | 27,8750 | 29,0827 | 1 | 28,6571 | 29,0826 | 3 |
| 44 | 28,6573 | 30,0450 | 2 | 29,4328 | 30,0449 | 2 |
| 45 | 29,4329 | 30,3690 | 2 | 30,1169 | 30,3689 | 2 |
| 46 | 30,1170 | 30,5359 | 2 | 30,3885 | 30,5358 | 2 |
| 47 | 30,3886 | 31,0956 | 3 | 30,6143 | 31,0955 | 3 |
| 48 | 30,6144 | 31,4098 | 0 | 31,2198 | 31,4097 | 2 |
| 49 | 31,2199 | 31,7338 | 2 | 31,5013 | 31,7337 | 3 |
| 50 | 31,5014 | 32,4211 | 0 | 32,0348 | 32,4210 | 3 |
| 51 | 32,0349 | 33,5732 | 2 | 32,8236 | 33,5731 | 3 |
| 52 | 32,8237 | 33,9888 | 0 | 33,6418 | 33,9887 | 2 |
| 53 | 33,6419 | 35,4125 | 0 | 34,7087 | 35,4124 | 6 |
| 54 | 34,7089 | 35,6482 | 0 | 35,4583 | 35,6481 | 2 |
| 55 | 35,4584 | 36,3126 | 3 | 35,6710 | 36,3125 | 4 |
| 56 | 35,6711 | 36,8035 | 2 | 36,4696 | 36,8034 | 5 |
| 57 | 36,4697 | 37,7428 | 0 | 37,0391 | 37,7427 | 3 |
| 58 | 37,0392 | 38,1880 | 0 | 37,9620 | 38,1879 | 2 |
| 59 | 37,9621 | 38,5021 | 0 | 38,3286 | 38,5021 | 3 |
| 60 | 38,3287 | 38,7247 | 2 | 38,5642 | 38,7246 | 3 |
| 61 | 38,5643 | 39,0749 | 0 | 38,9014 | 39,0748 | 2 |
| 62 | 38,9014 | 39,3433 | 3 | 39,1370 | 39,3432 | 3 |
| 63 | 39,1371 | 39,9946 | 4 | 39,6738 | 39,9945 | 5 |
| 64 | 39,6738 | 40,2499 | 0 | 40,0567 | 40,2498 | 2 |
| 65 | 40,0568 | 40,8030 | 0 | 40,4233 | 40,8029 | 2 |
| 66 | 40,4233 | 41,6245 | 1 | 41,3200 | 41,6244 | 2 |
| 67 | 41,3201 | 41,9158 | 0 | 41,7651 | 41,9157 | 2 |
| 68 | 41,7652 | 42,4067 | 0 | 41,9353 | 42,4066 | 4 |
| 69 | 41,9354 | 42,6882 | 2 | 42,4394 | 42,6881 | 4 |
| 70 | 42,4394 | 43,1431 | 3 | 42,8386 | 43,1430 | 3 |
| 71 | 42,8387 | 43,5555 | 0 | 43,3492 | 43,5554 | 3 |
| 72 | 43,3493 | 43,7290 | 0 | 43,6241 | 43,7289 | 2 |
| 73 | 43,6243 | 43,8403 | 0 | 43,7485 | 43,8402 | 3 |
| 74 | 43,7486 | 44,0988 | 0 | 43,8533 | 44,0987 | 3 |
| 75 | 43,8533 | 44,5276 | 5 | 44,2558 | 44,5275 | 3 |
| 76 | 44,2559 | 45,4833 | 0 | 45,3130 | 45,4832 | 2 |
| 77 | 45,3131 | 45,8171 | 2 | 45,5944 | 45,8170 | 5 |
| 78 | 45,5945 | 46,0724 | 3 | 45,9086 | 46,0723 | 1 |
| 79 | 45,9087 | 46,7957 | 0 | 46,2883 | 46,7956 | 1 |
| 80 | 46,2884 | 47,1001 | 2 | 46,8610 | 47,1000 | 3 |
| 81 | 46,8611 | 47,4437 | 2 | 47,1098 | 47,4436 | 3 |
| 82 | 47,1099 | 48,4158 | 2 | 47,7938 | 48,4157 | 5 |
| 83 | 47,7939 | 48,9067 | 3 | 48,4975 | 48,9066 | 2 |
| 84 | 48,4976 | 49,2798 | 3 | 48,9230 | 49,2797 | 2 |
| 85 | 48,9231 | 49,8460 | 2 | 48,5219 | 49,8459 | 2 |
| 86 | 49,5220 | 50,4712 | 3 | 49,8558 | 50,4711 | 4 |
| 87 | 49,8559 | 51,2337 | 0 | 50,4874 | 51,2337 | 3 |
| 88 | 50,4875 | 51,8720 | 0 | 51,2828 | 51,8719 | 4 |
| 89 | 51,2828 | 52,4218 | 3 | 51,8784 | 52,4217 | 2 |
| 90 | 51,8785 | 53,2269 | 0 | 52,5330 | 53,2269 | 3 |
| 91 | 52,5331 | 53,4888 | 2 | 53,2923 | 53,4887 | 3 |
| 92 | 53,2924 | 53,7899 | 2 | 53,5738 | 53,7898 | 2 |
| 93 | 53,5739 | 54,2840 | 6 | 53,8061 | 54,2840 | 3 |
| 94 | 53,8063 | 54,4543 | 1 | 54,2938 | 54,4542 | 2 |
| 95 | 54,2939 | 54,7914 | 2 | 54,5557 | 54,7913 | 4 |
| 96 | 54,5557 | 54,9845 | 0 | 54,8339 | 54,9844 | 4 |
| 97 | 54,8340 | 55,4034 | 0 | 55,1120 | 55,4033 | 4 |
| 98 | 55,1121 | 55,9500 | 3 | 55,6553 | 55,9499 | 5 |
| 99 | 55,6554 | 56,6439 | 2 | 56,4016 | 56,6438 | 5 |
| 100 | 56,4017 | 57,1610 | 0 | 56,7387 | 57,1409 | 0 |
| 101 | 56,7388 | 57,6094 | 2 | 57,1805 | 57,6093 | 2 |
| 102 | 57,1806 | 58,4374 | 2 | 57,8907 | 58,4373 | 1 |
| 103 | 57,8909 | 59,3244 | 0 | 59,0428 | 59,3243 | 3 |
| 104 | 59,0429 | 60,1000 | 3 | 59,5305 | 60,1000 | 3 |
| 105 | 59,5306 | 60,8168 | 0 | 60,1229 | 60,8167 | 0 |
| 106 | 60,1230 | 61,1866 | 2 | 60,8396 | 61,1866 | 5 |
| 107 | 60,8397 | 61,7529 | 0 | 61,4778 | 61,7528 | 0 |
| 108 | 61,4779 | 62,3485 | 2 | 61,9590 | 62,3484 | 3 |
| 109 | 61,9590 | 62,8231 | 0 | 62,4695 | 62,8230 | 3 |
| 110 | 32,4696 | 63,0980 | 0 | 62,9572 | 63,0979 | 0 |
| 111 | 62,9573 | 63,5497 | 2 | 63,2223 | 63,5496 | 2 |
| 112 | 63,2224 | 64,0210 | 2 | 63,6478 | 64,0209 | 3 |
| 113 | 63,6479 | 64,6821 | 0 | 64,1976 | 64,6820 | 5 |
| 114 | 64,1977 | 64,8850 | 0 | 64,7213 | 64,8849 | 0 |
| 115 | 64,7214 | 65,5102 | 2 | 64,9864 | 65,5101 | 3 |
| 116 | 64,9865 | 66,0076 | 1 | 65,6934 | 66,0075 | 1 |
| 117 | 65,6935 | 66,6753 | 0 | 66,2956 | 66,6752 | 3 |
| 118 | 66,2956 | 67,0190 | 2 | 66,6948 | 67,0189 | 4 |
| 119 | 66,6950 | 67,6146 | 0 | 67,0582 | 67,6145 | 3 |
| 120 | 67,0583 | 67,8437 | 0 | 37,7160 | 67,8437 | 4 |
| 121 | 67,7161 | 68,0107 | 0 | 67,8862 | 68,0106 | 3 |
| 122 | 67,8863 | 68,1710 | 3 | 68,0237 | 68,1709 | 3 |
| 123 | 68,0238 | 68,4067 | 0 | 68,1873 | 68,4066 | 3 |
| 124 | 68,1874 | 69,1169 | 0 | 68,9761 | 69,1168 | 3 |
| 125 | 68,9762 | 69,9220 | 0 | 69,4670 | 69,9219 | 4 |
| 126 | 69,4671 | 70,6257 | 2 | 70,0267 | 70,6256 | 4 |
| 127 | 70,0268 | 70,9072 | 0 | 70,7533 | 70,9071 | 3 |
| 128 | 70,7533 | 72,2196 | 0 | 71,7908 | 72,2195 | 1 |
| 129 | 71,7909 | 72,9789 | 2 | 72,3603 | 72,9788 | 2 |
| 130 | 72,3604 | 73,8593 | 0 | 73,4928 | 73,8593 | 4 |
| 131 | 73,4928 | 74,2423 | 0 | 74,1112 | 74,2422 | 4 |
| 132 | 74,1114 | 74,4976 | 0 | 74,3240 | 74,4975 | 1 |
| 133 | 74,3241 | 74,8739 | 3 | 74,6644 | 74,8739 | 1 |
| 134 | 74,6645 | 75,1914 | 0 | 75,0146 | 75,1913 | 2 |
| 135 | 75,0147 | 75,7347 | 0 | 75,0211 | 75,7346 | 2 |
| 136 | 75,0212 | 76,6740 | 0 | 76,5234 | 76,6739 | 4 |
| 137 | 76,5235 | 77,0340 | 0 | 76,6838 | 77,0340 | 3 |
| 138 | 76,6838 | 77,6461 | 0 | 77,4987 | 77,6460 | 2 |
| 139 | 77,4988 | 77,8654 | 0 | 77,7344 | 77,8653 | 3 |
| 140 | 77,7345 | 78,4054 | 0 | 78,2482 | 78,4053 | 4 |
| 141 | 78,4185 | 78,6574 | 0 | 78,4184 | 78,6573 | 3 |
| 142 | 80,3528 | 80,6277 | 0 | 80,3527 | 80,6276 | 2 |
| 143 | 81,2037 | 81,7405 | 0 | 81,2036 | 81,7404 | 0 |
| 144 | 82,2281 | 82,4736 | 0 | 82,2281 | 82,4735 | 0 |
| 145 | 85,1869 | 85,5600 | 0 | 85,1868 | 85,5599 | 4 |
| 146 | 85,7924 | 86,3651 | 0 | 85,7923 | 86,3650 | 0 |
| 147 | 90,0798 | 90,3908 | 0 | 90,0798 | 90,3907 | 0 |
| 148 | 90,4568 | 90,8195 | 0 | 90,4267 | 90,8194 | 0 |

HSSE-PDMS

2TF-SMPE

Figure S1. Overloaping chromatograms of *Hojiblanca* variety EVOO.

Figure S2. Overloaping chromatograms of *Picual* variety EVOO.

Figure S2. Overloaping chromatograms of *Picual* variety EVOO.

REFERENCES TABLE 3

[1] Choi HS, Kim MSL, Sawamura M. Constituents of the essential oil of Cnidium officinale Makino, a Korean medicinal plant. Flavour Fragr J 2002;17:49–53.

[2] Liang HY, Chen JY, Reeves M, Han BZ. Aromatic and sensorial profiles of young Cabernet Sauvignon wines fermented by different Chinese autochthonous Saccharomyces cerevisiae strains. Food Res Int 2013;51:855–65.

[3] National Center for Biotechnology Information. PubChem Compound Database. (2004) n.d. https://pubchem.ncbi.nlm.nih.gov/.Accessed September 9, 2019.

[4] Fan W, Qian MC. Characterization of aroma compounds of Chinese “Wuliangye” and “Jiannanchun” liquors by aroma extract dilution analysis. J Agric Food Chem 2006;54:2695–704.

[5] Chevance FFV, Farmer LJ. Release of volatile odor compounds from full-fat and reduced-fat frankfurters. J Agric Food Chem 1999;47:5161–8.

[6] Lukić I, Radeka S, Grozaj N, Staver M, Peršurić D. Changes in physico-chemical and volatile aroma compound composition of Gewürztraminer wine as a result of late and ice harvest. Food Chem 2016;196:1048–57.
